# Screening tumor stage-specific candidate neoantigens in thyroid adenocarcinoma using integrated exome and transcriptome sequencing

**DOI:** 10.3389/fimmu.2023.1187160

**Published:** 2023-10-03

**Authors:** Meng Jia, Jiawen Liang, Zhuyao Li, Ye Qin, Qianqian Li, Jianwei Wang, Xiubo Lu

**Affiliations:** ^1^ Department of Thyroid Surgery, The First Affiliated Hospital of Zhengzhou University, Zhengzhou, China; ^2^ Academy of Medical Science, Zhengzhou University, Zhengzhou, China; ^3^ School of Computer and Artificial Intelligence, Zhengzhou University, Zhengzhou, China

**Keywords:** thyroid carcinoma, neoantigens, machine learning, immune, prognosis

## Abstract

**Background:**

The incidence of thyroid carcinoma (THCA), the most common endocrine tumor, is continuously increasing worldwide. Although the overall prognosis of THCA is good, patients with distant metastases exhibit a mortality rate of 5-20%.

**Methods:**

To improve the diagnosis and overall prognosis of patients with thyroid cancer, we screened specific candidate neoantigen genes in early- and late-stage THCA by analyzing the transcriptome and somatic cell mutations in this study.

**Results:**

The top five early-stage neoantigen-related genes (NRGs) were G protein-coupled receptor 4 [*GPR4*], chondroitin sulfate proteoglycan 4 [*CSPG4*], teneurin transmembrane protein 1 [*TENM1*], protein S 1 [*PROS1*], and thymidine kinase 1 [*TK1*], whereas the top five late-stage NRGs were cadherin 6 [*CDH6*], semaphorin 6B [*SEMA6B*], dysferlin [*DYSF*], xenotropic and polytropic retrovirus receptor 1 [*XPR1*], and ABR activator of RhoGEF and GTPase [*ABR*]. Subsequently, we used machine learning models to verify their ability to screen NRGs and analyze the correlations among NRGs, immune cell types, and immune checkpoint regulators. The use of candidate antigen genes resulted in a better diagnostic model (the area under the curve [AUC] value of the early-stage group [0.979] was higher than that of the late-stage group [0.959]). Then, a prognostic model was constructed to predict NRG survival, and the 1-, 3- and 5-year AUC values were 0.83, 0.87, and 0.86, respectively, which were closely related to different immune cell types. Comparison of the expression trends and mutation frequencies of NRGs in multiple tumors revealed their potential for the development of broad spectrum therapeutic drugs.

**Conclusion:**

In conclusion, the candidate NRGs identified in this study could potentially be used as therapeutic targets and diagnostic biomarkers for the development of novel broad spectrum therapeutic agents.

## 1 Introduction

Thyroid carcinoma (THCA) begins in the thyroid gland. The main types of thyroid cancer include differentiated thyroid cancer (papillary, follicular, and Hürthle cells), medullary cancer, and anaplastic thyroid cancer (aggressive cancer) (1). Most thyroid cancers are differentiated thyroid cancers (DTC) (2) that develop from thyroid follicular cells (3). Thyroid cancer can be diagnosed at an early stage. Most early-stage thyroid cancers are diagnosed when neck lumps or nodules are noticed in the patient (4). In some cases, early thyroid cancers are detected when individuals undergo imaging tests, such as ultrasound or computed tomography scans, for other health problems (5). The prognosis of patients with thyroid cancer is better than that of patients with other cancer types. The 5-year relative survival rate of patients with localized or regional THCA is >90%, whereas that of patients with distant THCA varies according to the THCA type (6, 7).

The neoantigens of tumor-specific mutated genes are recognized by T cells and participate in the immune response of tumor cells (8–11). The antigenicity and immunogenicity of tumors depend on T cell immunoselection, in which tumor cells with strong tumor-specific mutant antigens are eliminated and those with weak (possibly mutated tumor antigens) or no (tumor cells mutated during antigen processing or presentation) antigens survive (10). Immunotherapies that enhance the ability of endogenous T cells to destroy cancer cells have demonstrated therapeutic efficacy in various malignancies, leading to the widespread application of neoantigens in clinical settings (10, 12). However, the clinical relevance of T cells in killing tumor cells remains unclear. Moreover, whether newly produced tumor-specific antigens play a protective or destructive role remains unknown (13). Recent technological innovations have facilitated the detection of tumor-specific mutations using whole exome sequencing (14, 15). To screen candidate neoantigens for tumor-specific mutations, the expression of host genes must be evaluated. Only recurrent mutations that are highly expressed in tumor cells and lowly expressed or even silent in normal cells can be considered candidate neoantigens (16).

In this study, we analyzed the functional, gene, and mutational differences between patients with early- and late-stage thyroid cancer versus normal thyroid cancer. A machine learning model was applied for further selection of features for diagnosis. The top five mutated genes were identified at each stage as potential neoantigens using the RFE method, and the prognostic characteristics of the selected 10 neoantigen-related genes (NRGs) were analyzed using a risk prediction model. The identified NRGs were effective in diagnosis and prognosis assessment. Therefore, NRGs may improve diagnostic accuracy and facilitate the development of novel targeted immunotherapy approaches to improve the outcomes of patients with THCA.

## 2 Materials and methods

### 2.1 Data preparation

We downloaded both whole exome sequencing and RNA-seq data of THCA from The Cancer Genome Atlas (TCGA) database (https://portal.gdc.cancer.gov/). Gene expression profiles (Level 3 data were downloaded from the TCGA data coordination center. This dataset showed the gene-level transcription estimates as log2(x+1) transformed RSEM-normalized counts. Clinical information of all patients, including the MNT stage and survival data, was also retrieved. Clinical information was obtained from 506 patients with THCA, of which 572 and 486 underwent transcriptome and exome sequencing, respectively.

### 2.2 Differentially expressed gene analysis

For RNA-seq analysis, we used 572 samples, including 59 normal tissue samples. Limma (17) (version 3.48.3 for R 4.2.3) was used to select the DEGs in early- and late-stage THCA compared to those in normal tissues. All genes with *p*< 0.05 and logFC beyond the 95% confidence interval were considered to be differentially expressed. We used the UpSetR package (version 1.4.0) to capture the overlapping genes of each group. PCA was used to analyze the distribution of each group. The Pheatmap package (version 1.0.12) was used to determine the expression of genes in the tumor (early and late stages) and normal groups. Kyoto Encyclopedia of Genes and Genomes (KEGG) analysis was used to analyze the relationship between differential genes and tumor pathways in the normal versus tumor group (early and late stages) and early versus late stage.

### 2.3 Cluster and function analyses

Genes that were overexpressed at any stage were selected as potential neoantigens. These genes are expressed at low levels or are silent in normal tissues, which makes the development of tumor-specific antigens safe and harmless. Visual analysis of the mutant profiles in patients with early and advanced thyroid cancer was performed using the maftools package (18) (version 2.8.05 for R 4.2.3). Furthermore, differential genes with high mutation frequencies were visualized in the early versus late group, and the mutation sites were integrated for candidate overexpressed genes. We mainly use corresponding R packages such as ggplot2 (version 3.4.2) to draw diagrams.

### 2.4 Screening of candidate neoantigen related genes

Neoantigens should only be present in patients and expressed at low levels in normal samples. Therefore, we downloaded neoantigens from The Cancer Immunome Atlas database (https://tcia.at/). The mutant samples were intersected with the neoantigen fragment genes and the differentially expressed upregulated genes were obtained using TCIA, and candidate NRGs were initially identified. Preliminary candidates for neoantigens in the early- and late-stage groups were identified using the Search Tool for the Retrieval of Interacting Genes/Proteins (https://string-db.org/) and protein–protein interaction (PPI) networks (19). We used Pearson correlations to analyze the associations between NRGs and constructed PPI networks using Cytoscape (version 3.8.2) (20). Boxplot (version 1.3.1) and other R packages are used to generate figures.

### 2.5 Construction of a diagnostic model

Candidate neoantigens are only overexpressed in the early or late stages of THCA; therefore, they can be considered potential therapeutic targets. To verify the feasibility of the preliminary screening of candidate neoantigens for the diagnostic model, we used caret package (version 6.0-94 for R 4.2.3, https://cran.r-project.org/web/packages/caret/index.html) with cross validation to screen feature combinations for NRG (21). mLR (nnet, version 7.3-18, https://cran.r-project.org/web/packages/nnet/index.html), Dtree (rpart, version 4.1.19, https://cran.r-project.org/web/packages/rpart/index.html), RF (randomForest, version 4.7-1.1, https://cran.r-project.org/web/packages/randomForest/index.html), and SVM (e1071, version 1.7-13, https://cran.r-project.org/web/packages/e1071/index.html), were used to construct four machine learning models. The area under the receiver operating characteristic (ROC) curve and 10 cross-prediction ROC curves were used to evaluate the feasibility of these NRGs. TSNAdb ((http://biopharm.zju.edu.cn/tsnadb/browse/)) used to analyze the mutant protein polypeptides to estimate their binding affinity for HLA alleles.

### 2.6 Immunoinfiltration analysis of NRGs

CIBERSORT (22), a calculation method used to quantify the immune cell fraction from RNA-seq data, was used to calculate the immune cell infiltration score in THCA. We analyzed the differences in early- and late-stage NRG immune ratios according to the immune cell type and determined the potential correlations between NRG expression and different types of infiltrating immune cells. Finally, we collected inhibitory immune checkpoints (chemokines, receptors, MHC, and immunostimulators) with therapeutic potential from a previous study (23)and determined their potential correlation with NRGs. Plots were generated with boxplot (version 1.3.1) and ggcorrplot (version 0.1.4) packages.

### 2.7 Construction of a prognostic model for neoantigen-associated genes

As host genes carrying these mutations are all overexpressed in patients with THCA, NRGs serve as potential diagnostic biomarkers or therapeutic targets. Next, we assessed the impact of these genes on patient outcomes. Based on the 10 gene signatures, the NRG score was calculated using the following formula: $\text{NRG score }=\overset{n}{\underset{i=1}{\sum }}coef\times exp_{i}$ , where exp indicates the expression level of NRGs. The median risk score was used to divide the patients into high- and low-risk groups.

### 2.8 Survival analysis

Kaplan–Meier survival analysis was performed using the survival (24) (version 3.5-5 for R 4.2.3). and survminer (25) (version 0.4.9) R packages. ROC curve was used to evaluate the prediction efficiency of genes for 1-, 3-, and 5-year survival with the R package, timeROC (26) (version 0.4), and log-rank test was used for comparison between groups. Wilcox test was used to compare the statistical differences between two groups. *p<* 0.05 was considered to be statistically significant. R, version 4.2.3 (http://www.r-project.org) is the main software environment for our statistical operation and graphics.

### 2.9 Comparison of candidate NRG expression levels

To investigate whether the identified NRGs were specific to THCA or common to other types of cancer, we used the GSCALite (27) (http://bioinfo.life.hust.edu.cn/web/GSCALite/) to compare the corresponding expression patterns in other cancer datasets from TCGA database (27). Comparison of NRG expression levels across tumors revealed the potential of shared neoantigens to act as broad spectrum therapeutic agents.

## 3 Results

### 3.1 Sample demographic statistics

Basic clinical information, including sex, age, TNM stage, and vital status, of the patients was shown in **Table 1**. The number of patients older than 55 years with late-stage THCA was higher than of patients in the early-stage (*p*< 0.001). More T_1_ stage patients were in the early stage (*p*< 0.001), and most patients were alive (*p* = 0.001). However, no significant difference in sex or TNM stage was observed between the early- and late-stage THCA groups (*p* = 0.093, 0.097, and 0.978, respectively; **Table 1**).

**Table 1:** Overview of thyroid carcinoma (THCA) early- and late-stage clinical data from The Cancer Genome Atlas (TCGA) database.

Clinical characteristics	Stage		χ^2^	*p*
	Early (284)	Late (219)		
Gender			2.816	0.093
Male (136)	68	68		
Female 367)	216	151		
Age			124.000	<0.001
<55 (337)	249	88		
≥55 (166)	35	131		
T			89.121	<0.001
T_1_(141)	121	20		
T_2_(166)	96	70		
T_3_(171)+T4(23)+TX(2)	67	129		
N			2.762	0.097
N_0_(229)	139	90		
N_1_(225)+NX(49)	145	129		
M			0.001	0.978
M_0_(281)	158	123		
M_1_(9)+M_X_(213)	126	96		
Vital status			11.210	0.001
Alive (487)	282	205		
Dead (16)	2	14		

### 3.2 Distribution of DEGs

**Figure 1:**
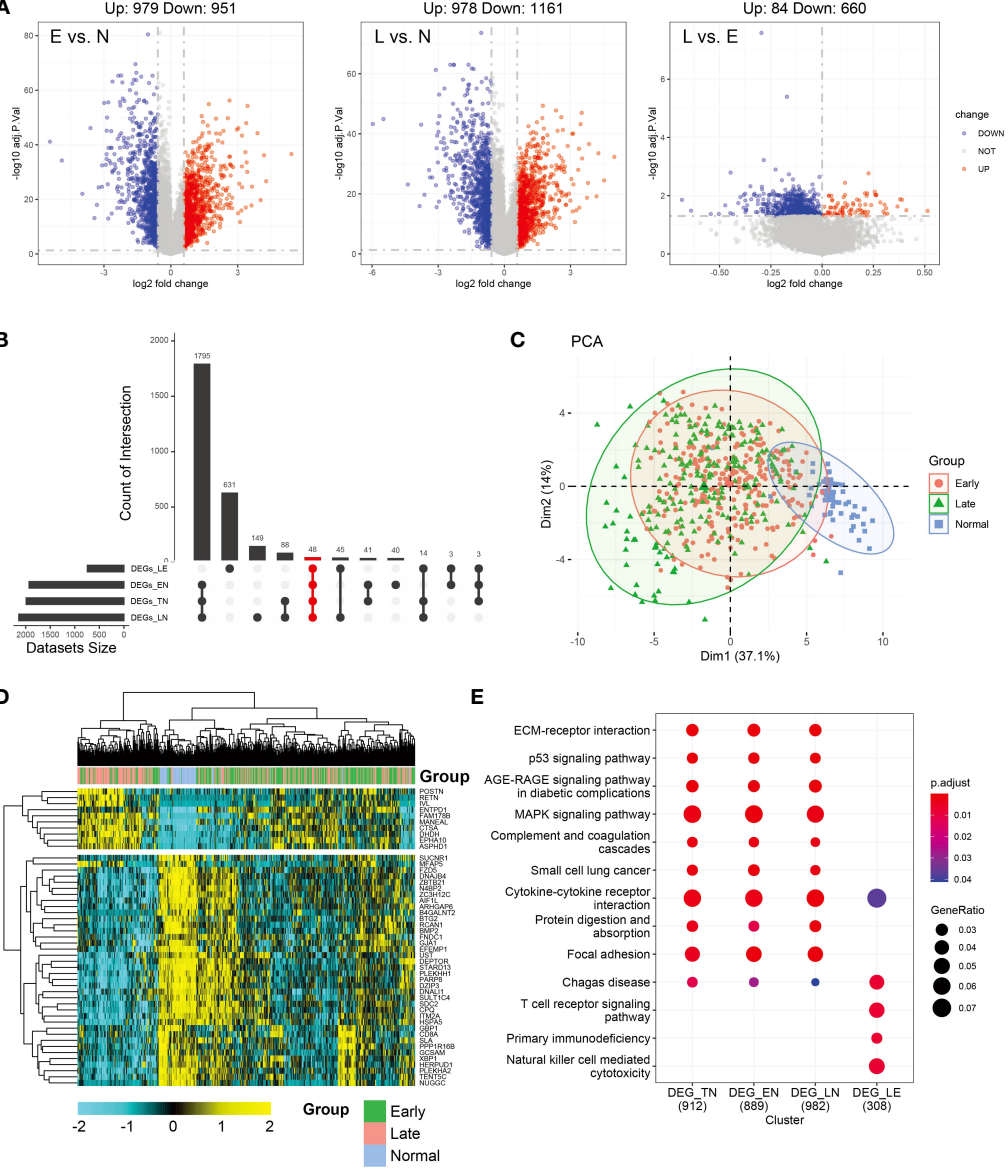
Analysis of differentially expressed genes (DEGs) in thyroid carcinoma (THCA) in normal and tumor tissues using The Cancer Genome Atlas (TCGA) data. **(A)** Volcano plot of DEGs of the two stages. The X axis represents the log2 fold-change, and the Y axis represents the negative log transformation of *p* values. Up- and downregulated genes are indicated by red and blue colors, respectively. Non-DEGs are indicated by grey color. **(B)** Distribution of samples in the count group of the database. **(C)** Intersections of DEGs of the two stages were used to cluster the samples. Normal, early-, and late-stage patients are indicated by blue, red, and green, respectively. **(D)** Heatmap of 47 genes between normal and tumor tissues. **(E)** Most significant Kyoto Encyclopedia of Genes and Genomes (KEGG) enrichment across normal and tumor subgroup DEGs.

Differential analysis was conducted between the normal and other subgroups (tumor group, early, and late stages) and tumors in the early and late stages. As shown in **Figure 1A**, 1957 and 2112 DEGs achieved based on 2-fold change from normal to early and late stages, respectively. Upregulated and downregulated genes tended to be balanced when comparing tumor patients with normal controls. However, in the comparison of the early and late stages, DEGs were predominantly upregulated. In total, 979 upregulated and 951 downregulated genes were identified in the early-stage group and 978 upregulated and 1161 downregulated genes were identified in the late-stage group. Moreover, 84 genes were upregulated and 660 genes were downregulated in the early stage compared to the late stage. However, in the normal group, compared to the tumor group, the upregulated and downregulated genes were balanced (971,1018; **Supplementary Figure 1A**). Further analysis of the upset plot revealed 48 genes shared between normal controls and patients with tumors (early stage, late stage, and tumor group) and early versus late stage (**Figure 1B**). As shown in **Figure 1C**, the tumor and normal samples were separated into different groups based on the DEGs. There are also some distinctions between the early and late stages. The heatmap was used to visualize gene expression patterns across all samples but could not clearly distinguish between early- and late-stage samples. The heatmap result was consistent with the PCA finding that tumor samples showed diverse patterns compared to normal samples (**Figure 1D**). KEGG enrichment analysis of DEGs was similar and was mainly related to tumor pathways. These mainly include the MAPK signaling pathway, cytokine-cytokine receptor interaction, focal adhesion, and other pathways. However, in the early and late subgroups, significantly enriched KEGG domains of DEGs were related to immunity, mainly Chagas disease, T cell receptor signaling pathway, primary immunodeficiency, and natural killer cell-mediated cytotoxicity. Moreover, Cytokine-cytokine receptor and Chagas disease were common pathways in the normal versus tumor subgroups and early versus late subgroups (**Figure 1E**). The differential genes and functions in the early and late stages of tumors corresponding to the normal group were not obvious; however, the differential genes in the early and late stages mainly played a role in immune function.

### 3.3 Neoantigen burden in early- and late-stage samples

**Figure 2:**
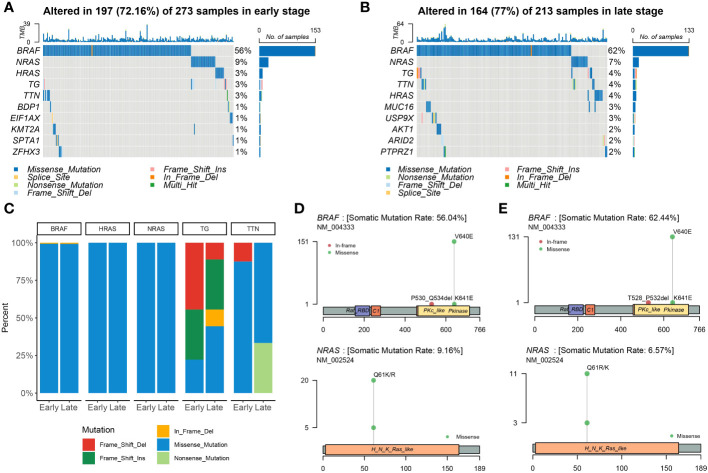
Landscape of mutations in THCA. Color-coded matrix of individual mutations in the top 10 most mutated genes in early **(A)**- and late **(B)**-stage THCA, indicating the number of recurrent mutations in THCA using TCGA data. **(C)** Stacked bar plot shows the fraction of variant types in the early and late stages. Mutation spots on BRAF and NRAS proteins in early **(D)** and late **(E)** stages. The number of mutations in each spot is shown on the Y axis. Mutation types are colored according to the legend.

At the beginning of the whole exome sequencing data analysis, we briefly summarized the mutation profile at the gene level. Analysis of early and late stages revealed that the missense mutation was the dominant variant (**Figures 2A, B**). At the gene level, the most frequently mutated genes in the early stages were *BRAF* (56%), *NRAS* (9%), *HRAS* (3%), *TG* (3%), and *TTN* (3%) (**Figure 2A**). The most frequently mutated genes in the late stage were *BRAF* (62%), *NRAS* (7%), *TG* (4%), *TTN* (4%), *HRAS* (4%), *MUC16* (3%), and *USP9X* (3%; **Figure 2B**). The mutation patterns of the mutated genes differed significantly between the early and late stages. We then examined all somatic mutations within the top 5% ranking by frequency; these were selected in early stage and late-stage patients using stacked bar plots. Early and late stages mainly involved missense mutations; *BRAF*, *HRAS*, and *NRAS* were the missense mutations in THCA. We also observed frame mutations in TG and nonsense mutations in TTN in the late-stage group (**Figure 2C**). *BRAF* and *NRAS* are proto-oncogenes, and their mutation spots had only one site (**Figures 2D, E**). We noted that two conserved domains of BRAF were mutated in the early- and late-stage groups and that these two domains were identical. The Pkinase and PKc-like domains had a missense mutation from V640E to K641E in both stages; however, the P530_Q534del event occurred in early thyroid cancer and the T528_P532del event occurred in late thyroid cancer. *BRAF*, *NRAS*, *TG*, *TTN*, and *HRAS* were the most frequently mutated genes in the early and late stages of the tumor; however, other genes were found to be more frequently mutated in the late stage.

### 3.4 Screening of candidate neoantigens

**Figure 3:**
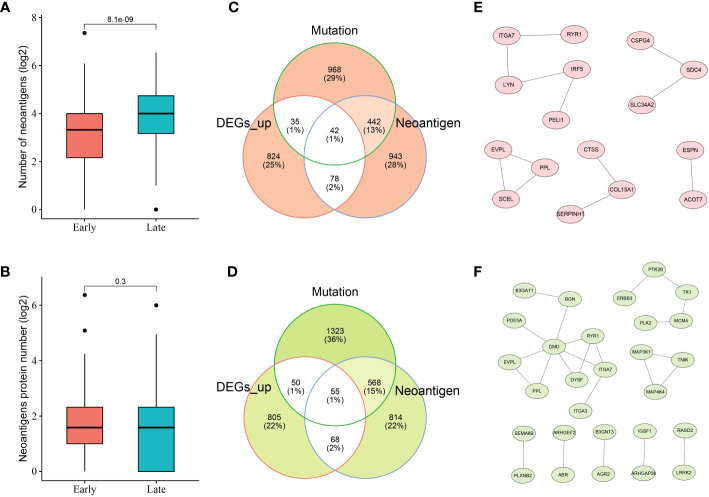
Neoantigen-related genes (NRGs) in THCA. Numbers of neoantigens **(A)** and associated neoantigen proteins **(B)** in early- and late-stage patients with THCA. Venn diagram indicating the 42 and 55 candidate neoantigen genes screened in normal versus early stage **(C)** and normal versus late stage **(D)** patients with thyroid cancer, respectively. The protein–protein interaction (PPI) networks of candidate neoantigen genes in early **(E)**- and late **(F)**-stage patients with THCA were found using the Search Tool for the Retrieval of Interacting Genes/Proteins (STRING) data.

Given the crucial role of NRGs in THCA, candidate neoantigens were screened and functional analyses were performed in this study. As shown in **Figures 3A, B**, we first screened the number of neoantigens in the early and late stages and then analyzed the number of associated neoantigen proteins. The results showed that The number of neoantigens in the late stage was significantly higher than that in the early stage. However, the number of neoantigen proteins tended to be balanced between early- and late-stage patients. To further screen the number of candidate neoantigens, Venn diagrams were used to screen the number of candidate neoantigens in normal versus other tumor subgroups (early and late stages; **Figures 3C, D**). Moreover, 42 neoantigens were identified in the early stage compared to the normal group (**Figure 3C**), and 55 neoantigens were identified in the late stage compared to the normal group (**Figure 3D**). Using the PPI network diagram, we identified 16 genes that interacted with the candidate neoantigens in the early stage (**Figure 3E**) and 28 genes that interacted with the candidate neoantigens in the late stage (**Figure 3F**). GO function analysis was performed according to the key factors of NRGs in THCA, which were mainly involved in the cAMP-mediated signaling pathway (**Supplementary Figure 2**). We preliminarily screened a number of candidate neoantigens using TCGA; however, the specific functions still need to be used for diagnostic model construction.

### 3.5 Diagnostic model construction

**Figure 4:**
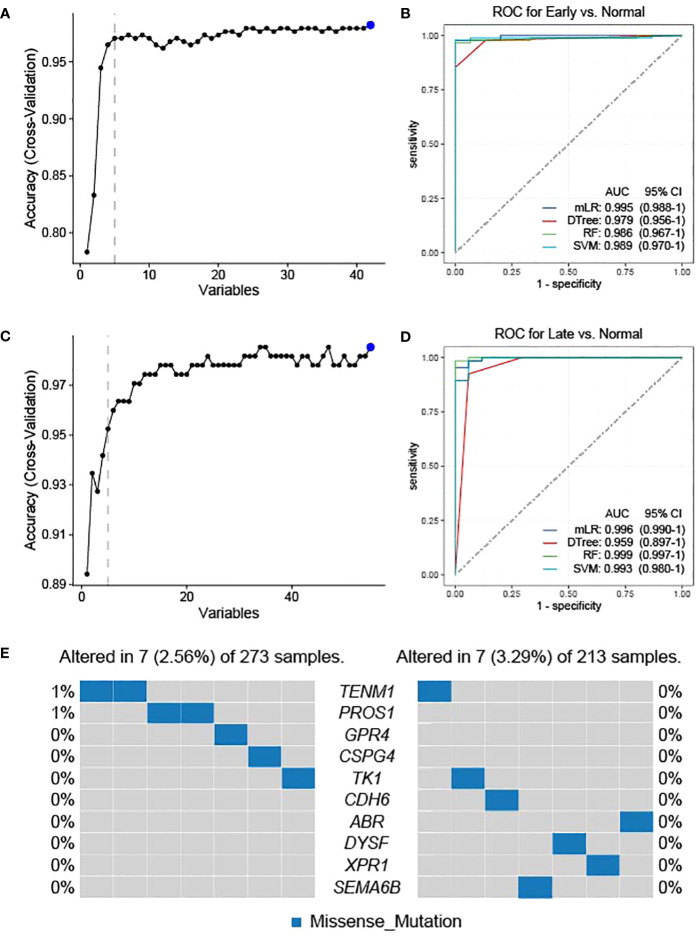
Feature selection and diagnostic model construction. Early **(A)**- and late **(C)**-stage feature filtering using the RFE method. Receiver operating characteristic (ROC) curve of 10-fold cross-prediction performed using four machine learning models in NRGs of normal versus early **(B)**- and late **(D)**-stage THCA. **(E)** Mutation frequencies of candidate neoantigens encoding variants in THCA.

As the candidate neoantigens are tumor-specific and the host genes are overexpressed in tumor tissues, host genes may also be used as diagnostic markers. Based on the preliminary screening of 42 genes in the normal versus early stage group, we used cross-validation to further screen the top 5 NRGs in the early versus normal stage (**Figure 4A**). The five NRGs identified were G protein-coupled receptor 4, chondroitin sulfate proteoglycan 4 [*CSPG4*], teneurin transmembrane protein 1 [*TENM1*], protein S [*PROS1*], and thymidine kinase 1 [*TK1*] (**Supplementary Table 1**). We then used the five NRGs to construct the area under the ROC curve using four machine learning models (mLR, Dtree, RF, and SVM); the lowest predictive value was 0.979 in the normal versus early stages (**Figure 4B**). Similarly, we preliminarily identified the top 5 NRGs (cadherin 6 [*CDH6*], semaphorin 6B [*SEMA6B*], dysferlin [*DYSF*], xenotropic and polytropic retrovirus receptor 1 [*XPR1*], and *ABR*) from 55 candidate neoantigens in late versus normal stages using cross-validation (**Figure 4C**; **Supplementary Table 1**). Similarly, the area under the curve (AUC) values of mLR, Dtree, RF, and SVM were 0.996, 0.959, 0.999, and 0.993, respectively, based on the ROC curves of the five NRGs selected from normal and late stages (**Figure 4D**). We further analyzed the mutation frequency of these 10 NRGs in a normal comparison of tumor stages (early and late). Frequencies of *ENM1* and *PROS1* mutations in the normal and early groups each accounted for 1%, whereas the other NRGs had no mutation frequency. In addition, no mutation frequency was observed in the 10 NRGs in the late stage of the normal contrast (**Figure 4E**). The results of TSNAdb (http://biopharm.zju.edu.cn/tsnadb/browse/) were used to predict candidate HLA neoantigen peptide fragments based on the NRGs (shown in **Supplementary Table 2**). The prediction used NetMHCpan4.0 reveals a strong binding affinity between GPR4, TENM1, and ABR with MHC molecules. Similarly, predictions using NetMHCpan2.8 found that TK1 and XPR1 have strong binding affinities with MHC. This data suggests the potential for these candidate NRGs to develop into new antigens, although further experimentation is required for confirmation.

### 3.6 Immunological correlation analysis of NRGs

**Figure 5:**
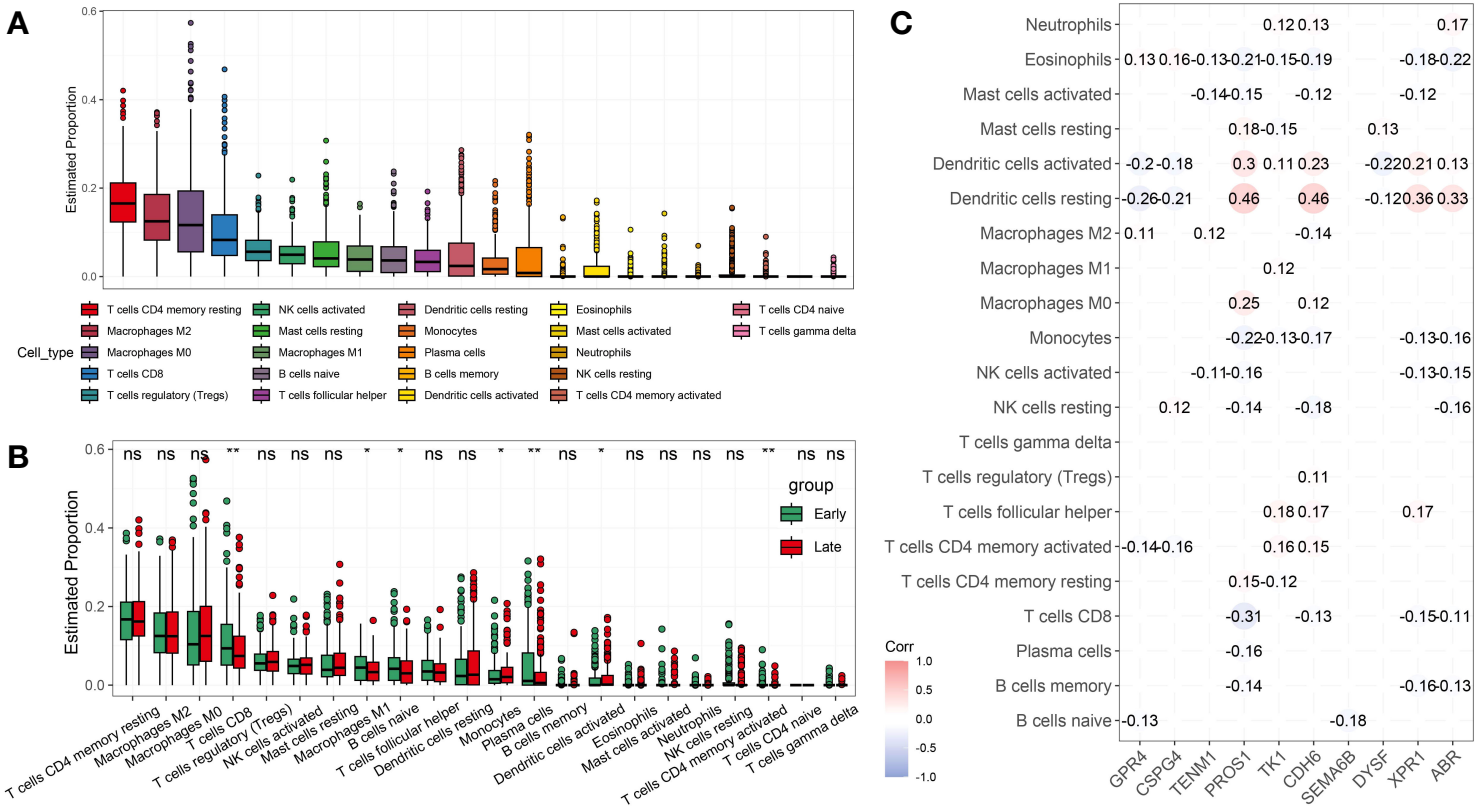
Analysis of candidate neoantigens among different immune subtypes. **(A)** Boxplot shows the proportion of the 22 types of immune cells in patients with THCA. Box plot **(B)** and correlation analysis **(C)** were used to analyze the association between NRGs and immune infiltrating cells in early- and late-stage THCA. *p< 0.05; **p< 0.01; ns, not significant.

**Figure 6:**
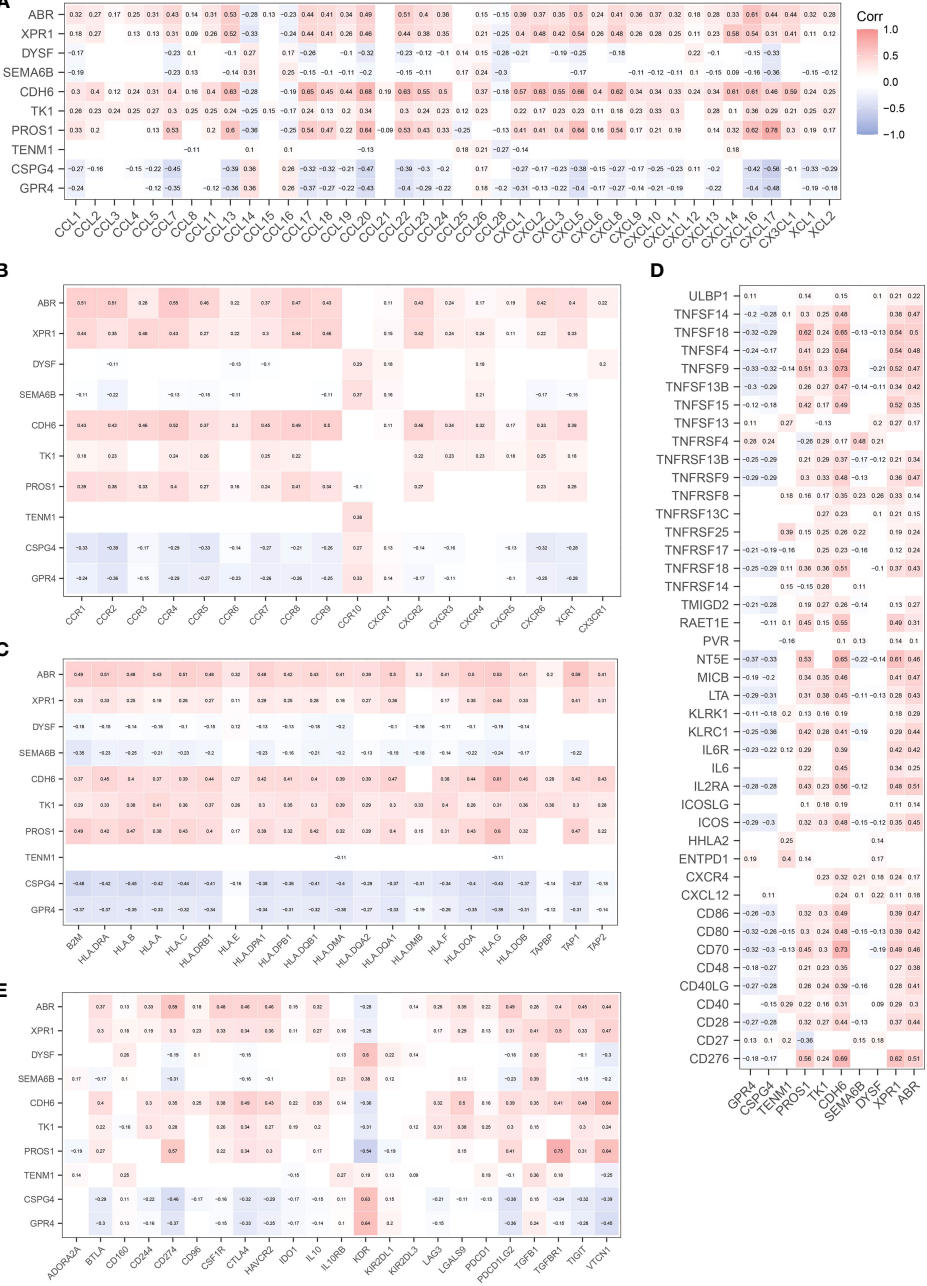
Correlation between candidate antigen genes and regulatory factors of relevant immune checkpoints. Chemokines **(A)**. Chemokine Receptors **(B)**. MHC Molecules **(C)**. Immunostimulators **(D)**. Immunoinhibitors **(E)**.

CIBERSORT (22) was used to analyze the effect of NRGs on the recruitment of immune cells to the recruitment of immune cells. We first analyzed the recruitment of immune cells to thyroid tissue, as shown in **Figure 5A**. The main immune cells recruited to the thyroid tissue were T cells CD4 memory resting, macrophages M2, macrophages M0, T cells CD8, and T cells regulatory (Tregs). We further investigated whether the recruitment of immune cells in the early and late groups differed from the recruitment of thyroid tissue described above. Macrophage M1 and naïve B cells, monocytes, and activated dendritic cells were differentially expressed in the early and late groups (*p*< 0.05), what’s more, T cells CD8, Plasma cells, and CD4 memory activated immune cells were significantly differentially expressed in the early and late groups (*p*< 0.01; **Figure 5B**). We further analyzed the correlation between NRGs and the type of immune infiltration, and the results are shown in **Figure 5C**. The types of immune infiltrates of *PROS1*, *CDH6*, *XPR1*, and *ARB* neoantigens were similar and were mainly positively correlated with dendritic cell activation and resting dendritic cells, and negatively correlated with Eosinophils and Monocytes. Most NRGs were negatively correlated with activated mast cells, NK cells activated, T cells CD8, B cells memory, and other immune cells. The results showed that the NRGs risk groups strongly correlated with different immune cell types. We also analyzed the immunological profiles of candidate neoantigen genes. Our data suggest that most genes were associated with immunostimulatory factors (**Figure 6**). *ABR*, *CPR1*, *CDH6*, *TK1*, and *PROS1* were positively correlated, whereas *CSPG4* and *GRPR4* were negatively correlated with immune checkpoint chemokines, chemokine receptors, MHC molecules, immune stimulators, and inhibitors.

### 3.7 Construction of a prognostic model for NRGs

**Figure 7:**
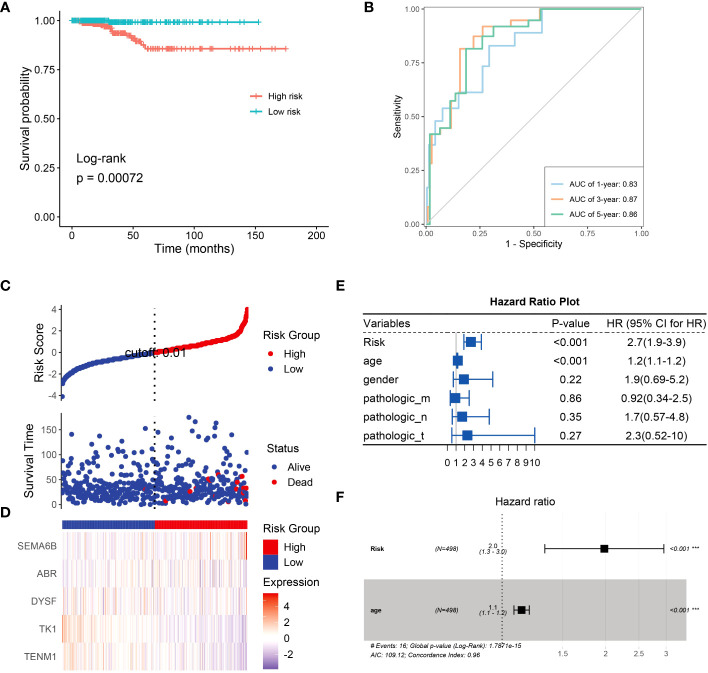
Univariate analysis using NRGs and prognostic model construction. **(A)** Risk model survival curves for NRGs. **(B)** Time-dependent ROC curves of NRGs to evaluate the performance of the prediction model. NRG expression levels in the high- and low-risk groups were assessed using the **(C)** risk score distribution and **(D)** heatmap. **(E)** Univariate analysis based on risk, age, gender, and TNM stage. **(F)** Univariate analysis based on age.

Next, we constructed a risk model to understand the effect of NRG expression on the prognosis of thyroid cancer and to evaluate the potential of BRGs as diagnostic markers. Univariate analysis based on the NRGs revealed that *SEMA6B* (*p*< 0.05, hazard ratio [HR] = 1.7) and *TENM1* (*p*< 0.05, HR = 0.53) were independent prognostic factors (**Supplementary Figure 3**). We successfully constructed a prognostic model using 10 candidate neoantigen genes. The NRGs score (risk = -0.5957*exp2$TENM1-0.9129*exp2$TK1-0.4365*exp2$DYSF+0.4896*exp2$ABR+0.7024*exp2$SEMA6B) was calculated using the formula, and the high- and low-risk groups were obtained. Furthermore, we found significant differences between the two groups by constructing a high-low-risk model; the prognosis of the high-risk group was significantly worse than that of the low-risk group (*p* = 0.00072; **Figure 7A**). The ROC curve showed that the AUC values were 0.83, 0.87, and 0.86 at 1, 3, and 5 years, respectively (**Figure 7B**). Almost all patients who died belonged to the high-risk group (**Figure 7C**). Heatmap analysis showed that *TK1* and *TENM1* expression levels were low in the high-risk group and high in the low-risk group. Genes with high expression in the high-risk group and low expression in the low-risk group included SEMA6B, ABR, and DYSF (**Figure 7D**). We then analyzed the clinical prediction model of NRGs by integrating the clinical characteristics of patients in the TCGA database. The results showed that the independent prognostic factors in the univariate analysis were risk (*p*< 0.001, HR = 2.7) and age (*p*< 0.001, HR = 1.2; **Figure 7E**). Multivariate analysis revealed that the risk (*p*< 0.001, HR = 2.0) and age (*p*< 0.001, HR = 1.0) were independent prognostic factors (**Figure 7F**). Therefore, we believe that NRGs can predict survival outcomes.

### 3.8 Analysis and comparison of NRG expression levels and mutation frequencies in various tumors

**Figure 8:**
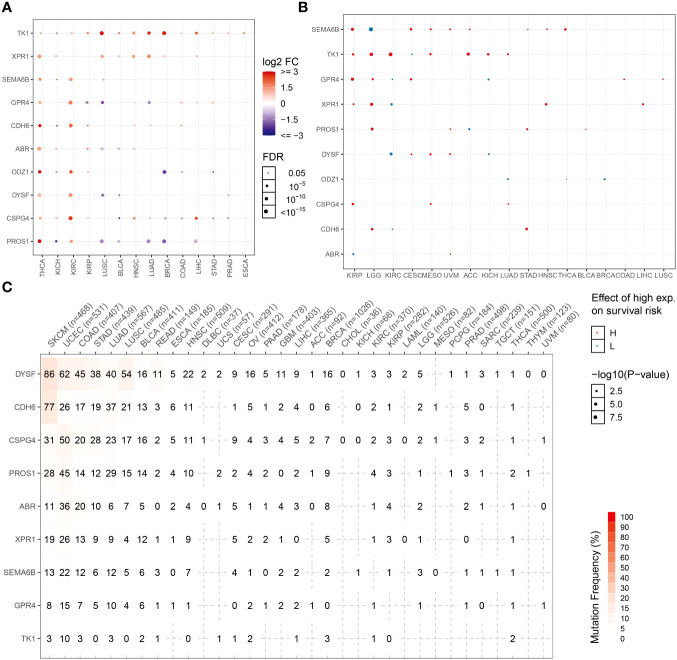
NRG expression levels and mutation frequencies in multiple tumors. Differential expression **(A)** and survival difference **(B)** analyses of NRGs in multiple tumors. **(C)** Mutation frequencies of NRGs in different tumor samples.

Expression patterns of NRGs in different tumor tissues were determined using the TCGA database. The candidate NRGs were all highly expressed in thyroid cancer tissues, and candidate neoantigen genes, such as *CDH6*, *ODZ1*, and *PROS1*, were significantly differentially expressed in thyroid cancers. *TK1* was expressed at low levels in KICH but highly expressed in 13 other tumor types (**Figure 8A**). To analyze the differential expression of NRGs, we examined the differential survival of NRGs in multiple tumors. As shown in **Figure 8B**, candidate neoantigen *SEMA6B* was associated with a worse prognosis, and the difference was obvious in the high-expression group of THCA. However, *ODZ1* expression was associated with a poor prognosis in the low THCA expression group. Candidate neoantigens, *TK1* and *XPR1*, were associated with poor prognosis in the high expression group for most tumors. NRGs mutate repeatedly in most tumors, especially skin cutaneous melanoma and uterine corpus endometrial carcinoma (**Figure 8C**). However, the frequency of NRG mutations is relatively low in THCA, possibly related to the favorable prognosis of thyroid tumors.

### 3.9 Analysis of functionally related drugs of candidate neoantigen genes

**Figure 9:**
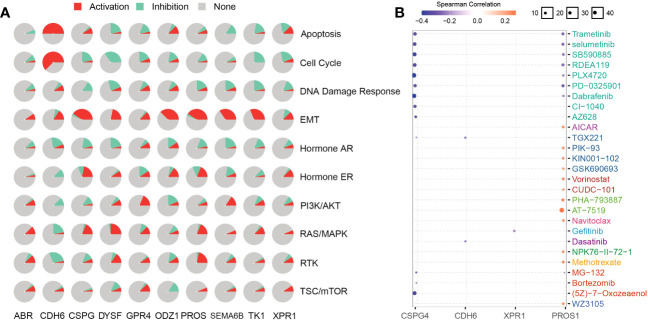
NRG functions and related drug analysis. **(A)** Activities of biological pathways identified using NRGs were analyzed using the GSCA database. Red and azure colors represent the percentage of activation and inhibition, respectively. Non-activity is indicated by grey color. **(B)** NRG-related drugs were obtained using the GSCA database.

To analyze NRG-related drug functions, we performed NRG pathway function analysis. NRGs are involved in the activation of epithelial–mesenchymal transition (EMT) but inhibit the DNA damage response. *CDH6* plays an inhibitory role in related pathways but participates in the activation of apoptosis, EMT, and cell cycle (**Figure 9A**). Simultaneously, the candidate-associated neoantigens (*CSPG4*, *XPR1*, and *CDH6*) negatively correlated with the known drugs. However, positive and negative correlations between PROS1 and the drugs occurred simultaneously or alternately (**Figure 9B**). Through functional analysis of candidate antigen gene drugs, we found that antitumor drugs were involved in antigen presentation. Therefore, the screened NRGs can be used as therapeutic targets and diagnostic biomarkers for thyroid cancer.

## 4 Discussion

Thyroid cancer is the most common endocrine tumor and widespread cancer in the USA (2, 28). Most thyroid cancers arise from thyroid follicular cells (90%) and are well-differentiated (29, 30). Most tumors are categorized on histological grounds as papillary thyroid cancers or anaplastic thyroid cancers, the latter being associated with a worse prognosis (2, 31). The standard therapeutic approach for all thyroid cancers includes surgery, with radioactive iodine offered to patients with follicular cell-derived thyroid cancers (1, 32, 33). Several preclinical studies have suggested the potential of immunotherapy for the treatment of thyroid cancer (30, 34). Although this general approach is less developed in terms of clinical trial data, several ongoing immunotherapy trials exhibit great clinical potential. However, the efficacy of immunotherapy for specific THCA stages remains unclear (30, 35, 36).

To predict the potential of candidate neoantigen genes for THCA screening, we comprehensively analyzed the data from the TCGA database and combined them with TSNAdb to predict the HLA candidate neoantigen peptide fragments in this study. Our findings revealed that NRGs had predictive correlations with THCA in terms of gene mutation frequency, DEGs, and type of immune cell infiltration. Expression patterns of downregulated genes were significantly different between the early and late stages of THCA. DEG analysis further revealed that the tumor samples were significantly different from the normal samples (**Figure 1**). Analysis of the top ten DEGs in candidate patients with early- and late-stage cancer revealed that the mutated genes were mainly concentrated in missense mutations (**Figure 2**). Genomic instability can be used as a marker of malignant tumors and plays an important role in the development and progression of tumors (37, 38). It not only promotes the evolution of tumors but also assumes a high neoantigen load by tumor cells, which is recognized and localized by the immune system (39, 40). Therefore, we preliminarily screened DEGs and analyzed their mutation types to prepare candidate neoantigens in this study. The ROC curve, consisting of four machine learning models (mLR, Dtree, RF, and SVM), was established via the preliminary screening of candidate neoantigen genes in the early and late groups (AUC of the normal and early groups was > 0.979 and that of the normal and late groups was > 0.959). We believe that the screened NRGs can be used as novel diagnostic biomarkers for thyroid cancer (**Figure 3**). Because neoantigen loading can be used as a potential biomarker for tumor immunotherapy, the accurate and effective prediction of neoantigens as therapeutic targets may aid in the development of personalized cancer vaccines.

Vaccines are commonly used to prevent infections. Interestingly, vaccines, especially neoantigen-targeted vaccines, also show promise for personalized immunotherapy of cancers, such as hepatocellular carcinoma, melanoma, and epithelial ovarian cancer, in preclinical and clinical studies (41–43). Personalized vaccines are designed to trigger tumor-specific T cell responses against neoantigens to expand the endogenous repertoire of tumor-specific T cells and prevent “off-target” damage to non-tumor tissues (44, 45). Here, we analyzed immune cell infiltration in thyroid cancer and found that the majority of immune-infiltrating cells were T cells (**Figure 5A**). Furthermore, we compared the immune infiltration in patients with early versus late thyroid cancer and found that the expression levels of T cells CD8, plasma cells, and CD4 + memory-activated immune cells were significantly different (*p*< 0.01; **Figure 5B**). Further analysis of the infiltration of 10 candidate NRGs revealed that dendritic and T cells had different degrees of positive and negative correlations with multiple NRGs (**Figure 5C**). Studies have shown that dendritic cells are primarily involved in acquiring, processing, and presenting tumor-associated antigens on MHC molecules in the tumor microenvironment (TME), and provide costimulators and soluble factors to shape T cell responses (46). Peng et al. reported that dendritic cells participate in antigen presentation and mediate the activation and reactivation of tumor-specific T cells through the ability of endogenous T cell compartments to recognize peptide epitopes that display MHC on the surface of malignant tumor cells (10, 47). We identified NRGs whose neoantigen epitopes result from tumor-specific DNA alterations leading to the formation of new protein sequences that can be used for post-treatment immune memory via neoantigen-specific T-cell responses and prevention of cancer recurrence.

NRGs present a new technique for thyroid cancer immunotherapy. Here, we found that NRGs were expressed in patients with multiple cancers and were significantly correlated (**Figure 8A**). The expression trends and mutation frequencies of the THCA candidate neoantigens were similar to those in KIRC. Personalized mRNA vaccines based on neoantigens can be used in expression strategies for KIRC solid tumors (48). Candidate neoantigens TK1 and XPR1 were expressed in multiple tumors, and the prognosis was poor in the high expression group of most tumors (**Figure 8B**). Xie et al. reported that TK1 deactivation significantly inhibits the growth of prostate tumors and is closely related to cell cycle regulation (49). Yoko et al. demonstrated that XPR1-dependent phosphate effervescence leads to the toxic accumulation of intracellular phosphate, inducing growth arrest and apoptosis in ovarian clear cell carcinoma cells (50, 51). To determine the correlation between NRGs and targeted drugs, we used the open GSCA database to identify gene-related targeted drugs for further analysis (**Figure 9B**). CSPG4, a high-molecular-weight melanoma-associated antigen, is negatively associated with multiple targeted drugs, such as cell-surface proteoglycans. Elevated CSPG4 expression is observed in several aggressive tumors, including ovarian cancer (52), osteosarcoma (53), and triple-negative breast cancer (54). In addition, Egan et al. demonstrated that CSPG4 is a potential immunotherapeutic target for ATCs (55). Bortezomib, as a chemotherapy agent, can trigger immunogenic cell death, thereby promoting anti-tumor immunity (56), and our candidate antigen, CSPG4, was strongly associated with Bortezomib. Trametinib, selumetinib, and PD-0325901 are strongly associated with targeting candidate antigens CSPG4 and PROS1, as the more commonly used MEK inhibitors. Zheng et al. found that the MEK inhibitors combined with radiotherapy can enhance antitumor immunity, and offer a new treatment strategy for KRAS mutations in the tumor (57). Therefore, drugs targeting the candidate antigen, CSPG4, can potentially be used to treat thyroid cancers. In conclusion, a comparison of the expression levels of NRGs among various tumors and their survival analysis revealed that NRGs can predict other tumors, indicating their potential for immunotherapy. Therefore, NRGs can be used as molecular markers for patients with thyroid cancer and converted into antigen-related mRNA vaccines for immunotherapy.

In this study, we screened candidate neoantigens as diagnostic features and therapeutic targets and identified specific candidate neoantigen genes in early- and late-stage thyroid cancer by combining transcriptome and somatic cell mutations. Using machine learning models, we found that the identified candidate genes successfully predicted early- and late-stage thyroid cancer. Simultaneously, we constructed a prognostic model and conducted pan-cancer and targeted drug analyses. Our results revealed that some candidate genes potentially act as candidate neoantigens. Therefore, the candidate tumor-specific neoantigens identified in this study can be used for the personalized treatment of patients with various thyroid tumors.

## Data availability statement

The original contributions presented in the study are included in the article/**Supplementary Material**. Further inquiries can be directed to the corresponding author.

## Author contributions

Conceptualization and Methodology: JW and MJ; Writing – Original Draft Preparation and Formal Analysis: MJ and JL; Writing – Review and Editing: JL and ZL; Investigation: YQ and QL; Supervision and Project Administration: XL. All the authors have reviewed the manuscript. All authors contributed to the article and approved the submitted version.
