# Supplementary material for: Screening tumor stage-specific candidate neoantigens in thyroid adenocarcinoma using integrated exome and transcriptome sequencing
Source: Front Immunol. 2023 Oct 3;14:1187160. doi: 10.3389/fimmu.2023.1187160 (PMC10579579; doi:10.3389/fimmu.2023.1187160)
Supplement: Supplementary file 1 [file DataSheet_1.docx]

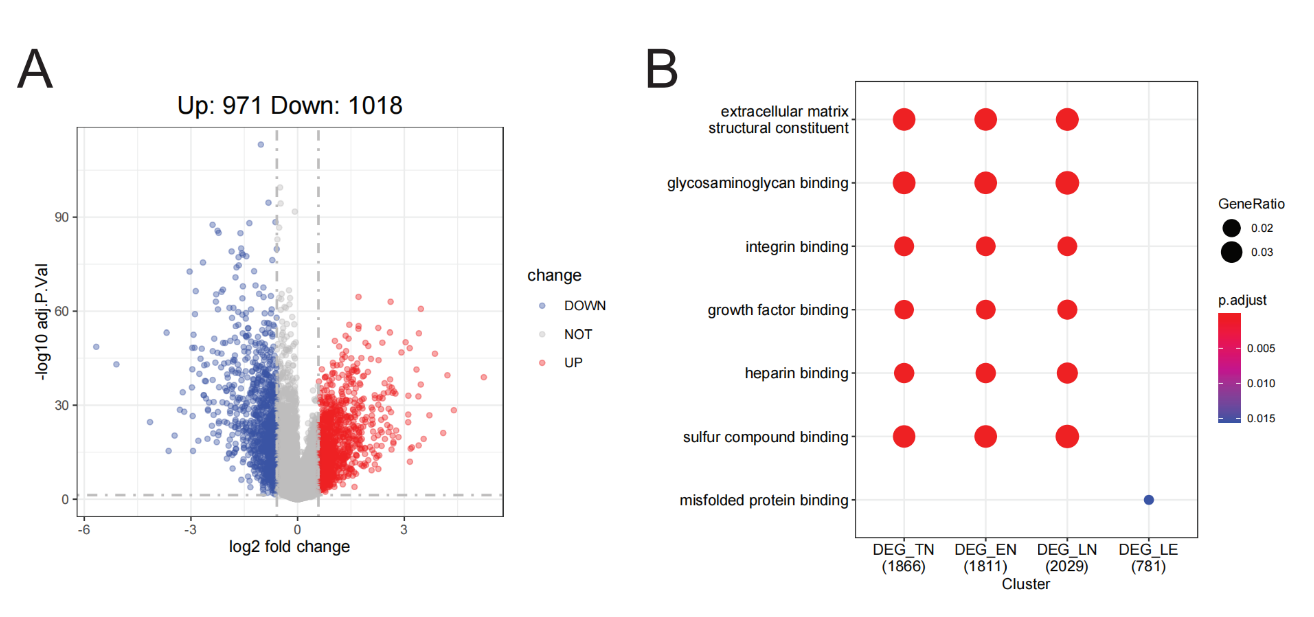


**Supplementary Figure 1.** Analysis of differential expression of THCA in early and late stage. (A). Volcano plot of DEGs between early and late stage. (B). The most significant GO enrichment across normal and tumor subgroups DEGs.


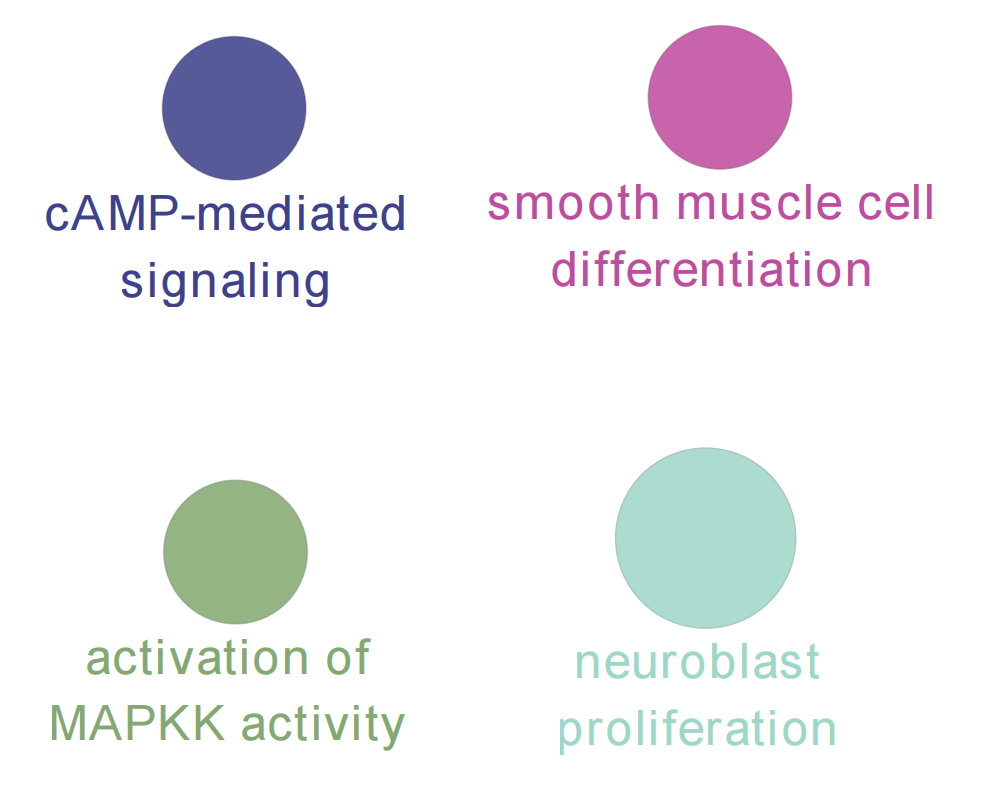


**Supplementary Figure 2.** GO functional enrichment analysis of differential genes was performed based on NRGs.


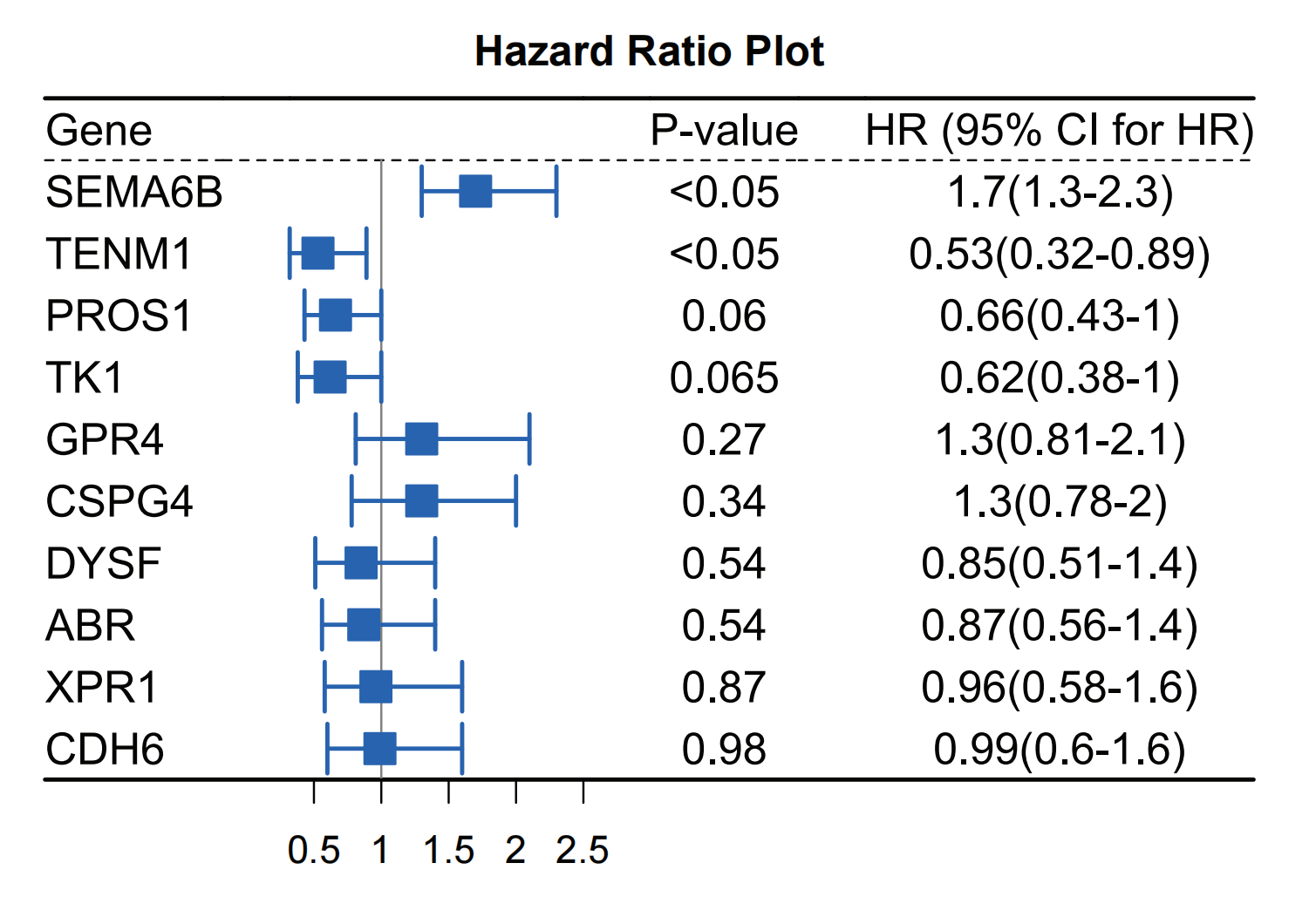


**Supplementary Figure 3.** Univariate analysis based on NRGs.


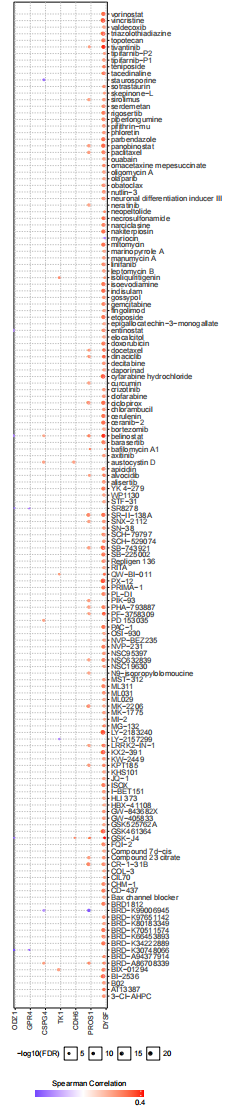


**Supplementary Figure 4.** NRGs related drugs were obtained based on GSCA database.

**Supplementary Table 1.** Neoantigen comparison between early and late stage

| Gene | Group |
| --- | --- |
| GPR4 | E-N |
| CSPG4 | E-N |
| TENM1 | E-N |
| PROS1 | E-N |
| TK1 | E-N |
| CDH6 | L-N |
| SEMA6B | L-N |
| DYSF | L-N |
| XPR1 | L-N |
| ABR | L-N |

Which neoantigen gene mutation number sample

**Supplementary Table 2.** HLA prediction of Candidate neoantigen peptide using TSNAdb

| Gene | Group | Mutation | Position in peptide | HLA allele | WT peptide | WT affinity (nM) | WT binding level | MT peptide | MT affinity (nM) | MT binding level | Frequency in the THCA |
| --- | --- | --- | --- | --- | --- | --- | --- | --- | --- | --- | --- |
| GPR4 | E-N | E157V | 9 | A*68:02 | NSAPLFHDE | 48560.98 | - | NSAPLFHDV | 24.37 | SB | 1/488 |
| GPR4 | E-N | E157V | 7 | B*53:01 | APLFHDELF | 23.76 | SB | APLFHDVLF | 14.13 | SB | 1/488 |
| CSPG4 | E-N | V1907M | 2 | A*02:01 | FVANGSSVA | 21372.79 | - | FMANGSSVA | 452.41 | WB | 1/488 |
| TENM1 | E-N | S1131F | 6 | B*44:02 | FEMDASNLGGW | 9.5 | SB | FEMDAFNLGGW | 55.79 | SB | 1/488 |
| TENM1 | E-N | P1221S | 4 | C*03:03 | RIFPSGNSV | 13.63 | SB | RIFSSGNSV | 215.18 | WB | 1/488 |
| TENM1 | E-N | S1131F | 2 | C*07:02 | ASNLGGWSL | 8384.65 | - | AFNLGGWSL | 301.08 | WB | 1/488 |
| TENM1 | E-N | P1221S | 2 | C*03:03 | FPSGNSVSI | 162.83 | WB | FSSGNSVSI | 6.51 | SB | 1/488 |
| PROS1 | E-N | K200I | 11 | C*03:03 | FVMLSNKKDCK | 24204.11 | - | FVMLSNKKDCI | 479.77 | WB | 1/488 |
| PROS2 | E-N | K200I | 2 | A*11:01 | CKDVDECSLK | 18284.78 | - | CIDVDECSLK | 246.47 | WB | 1/488 |
| PROS3 | E-N | Q571K | 1 | A*02:01 | QQSHLEFRV | 1154.09 | - | KQSHLEFRV | 169.68 | WB | 1/488 |
| TK1 | E-N | D83E | 9 | A*02:05 | ALPACLLRDV | 768.93 | - | ALPACLLREV | 105.28 | SB | 1/488 |
| TK1 | E-N | D83E | 4 | A*02:01 | LLRDVAQEAL | 428.07 | WB | LLREVAQEAL | 358.57 | WB | 1/488 |
| TK1 | E-N | D83E | 4 | A*02:05 | LLRDVAQEAL | 210.85 | WB | LLREVAQEAL | 225.11 | WB | 1/488 |
| TK1 | E-N | D83E | 9 | A*02:01 | ALPACLLRDV | 297.86 | WB | ALPACLLREV | 45.26 | SB | 1/488 |
| TK1 | E-N | K211T | 2 | A*11:01 | GKPGEAVAARK | 30493.72 | - | GTPGEAVAARK | 289.17 | WB | 1/488 |
| TK1 | E-N | D83E | 5 | A*02:01 | CLLRDVAQEAL | 267.19 | WB | CLLREVAQEAL | 353.17 | WB | 1/488 |
| TK1 | E-N | D83E | 5 | A*02:01 | CLLRDVAQEA | 276.37 | WB | CLLREVAQEA | 304.35 | WB | 1/488 |
| CDH6 | L-N | A349D | 7 | C*16:01 | YTLKVEASNPY | 86.17 | SB | YTLKVEDSNPY | 160.34 | WB | 1/488 |
| SEMA6B | L-N | G296R | 3 | B*55:01 | VPGDSHFYF | 4922.09 | - | VPRDSHFYF | 270.54 | WB | 1/488 |
| DYSF | L-N | R2041W | 1 | C*06:02 | RRFRWAIIL | 27.9 | SB | WRFRWAIIL | 213.16 | WB | 1/488 |
| XPR1 | L-N | E615K | 11 | A*11:01 | FVWNFFRLENE | 25447.24 | - | FVWNFFRLENK | 50.5 | SB | 1/488 |
| ABR | L-N | T126N | 4 | C*03:04 | TATTSQPVL | 216.18 | WB | TATNSQPVL | 111.18 | SB | 1/488 |

which neoantigen gene mutation number sample

HLA, human leukocyte antigen; WT, wild type; MT, mutant; The binding level ‘Strong’ indicates strong binding with IC50 < 150 nM, ‘Weak’ indicates weak binding with 150 nM < IC50 < 500 nM, ‘-’ indicates non-binding with IC50 > 500 nM
